# Studies of involvement of G-protein coupled receptor-3 in cannabidiol effects on inflammatory responses of mouse primary astrocytes and microglia

**DOI:** 10.1371/journal.pone.0251677

**Published:** 2021-05-13

**Authors:** Jun Wu, Nu Chen, Yongqing Liu, Grzegorz Godlewski, Henry J. Kaplan, Sarah H. Shrader, Zhao-Hui Song, Hui Shao

**Affiliations:** 1 Department of Ophthalmology and Visual Sciences, Kentucky Lions Eye Center, University of Louisville, School of Medicine, Louisville, Kentucky, United States of America; 2 Department of Ophthalmology, Union Hospital, Tongji Medical College, Huazhong University of Science and Technology, Wuhan, P. R. China; 3 Tianjin Key Laboratory of Retinal Functions and Diseases, Eye Institute and School of Optometry, Tianjin Medical University Eye Hospital, Tianjin, P.R. China; 4 Department of Medicine-Oncology, University of Louisville, School of Medicine, Louisville, Kentucky, United States of America; 5 Laboratory of Physiologic Studies, National Institute on Alcohol Abuse and Alcoholism of the National Institutes of Health, Bethesda, Maryland, United States of America; 6 Department of Pharmacology and Toxicology, University of Louisville, School of Medicine, Louisville, Kentucky, United States of America; University of Texas Medical Branch at Galveston, UNITED STATES

## Abstract

Cannabidiol (CBD) exhibits anti-inflammatory and neuroprotective properties and is suggested to be effective in the pre-clinical and clinical treatment of illnesses of the central nervous system (CNS). Two major types of CNS glial cells, astrocytes and microglia, play critical roles in the development and pathogenesis of CNS diseases. However, the mechanisms by which CBD plays an anti-inflammatory and neuroprotective role for these glial cells have not been fully elucidated. In this study, we examined the effects of CBD on the inflammatory response of mouse primary astrocytes and microglia. We also investigated whether the effect of CBD on cytokine release is mediated by the G protein coupled receptor 3 (GPR3), which was recently identified as a novel receptor for CBD. Our results showed that CBD inhibited inflammatory responses of astrocytes and microglia stimulated with lipopolysaccharide (LPS), a Toll-like receptor 4 (TLR4) ligand in vitro and in vivo. In addition, CBD reduced the phosphorylation of STAT3 and NF-κB signaling pathways in LPS-stimulated astrocytes. However, the inhibitory effect of CBD on pro-inflammatory cytokine production was independent of GPR3 expression in both types of glial cells. Thus, although CBD is effective in ameliorating the activation of astrocytes and microglia, its mechanism of action still requires further study. Our data support the concept that CBD may have therapeutic potential for neurological disorders that involve neuroinflammation.

## Introduction

Cannabidiol (CBD) is the major non-intoxicating component of cannabis [1]. Bolstered by its non-psychoactive nature, CBD has a wide range of potential therapeutic uses in the treatment of epilepsy [2], pain [3], anxiety disorders [4], cancer [5], inflammatory diseases [6], and neurodegenerative or neuropsychiatric diseases [7]. Preclinical evidence suggests that CBD is an immune-modulating agent that affects T cells [8, 9], B cells [10], macrophages [11] and microglia cells [12, 13], resulting in an overall reduction in pro-inflammatory cytokine expression and an increase in anti-inflammatory cytokines. Clinical trials involving CBD have indicated its efficacy for the treatment of multiple sclerosis (MS) [14] and inflammatory bowel disease [15, 16]. Although several signaling pathways and related targets of CBD involved in neuroprotection have been discovered such as peroxisome proliferator-activated receptor γ (PPARγ) [17], Wnt/β-catenin [18], NF-κB, and PI3K/Akt [19], its mechanism of anti-inflammatory action on astroglia remains unclear.

Astrocytes are the most abundant and one of the most important glial cell populations in the CNS. They regulate the blood-brain barrier (BBB), neuronal metabolism, electrical transmission, CNS development and repair [20, 21]. It is also known that astrocytes are involved in neuroinflammation [20, 21]. Previously, CBD was found to inhibit β–amyloid (Aβ)-induced inflammatory mediators (TNF-α, S100B, and IL-1) from rat primary astrocytes [17]. However, the effects of CBD on astrocyte release of IL-6, a cytokine that plays important roles in neuroinflammation, and lipopolysaccharide (LPS), a toll-like receptor 4 (TLR4) ligand [22], have not been examined.

G protein coupled receptor 3 (GPR3) is a member of the G protein-coupled receptor family of transmembrane receptors and belongs to a family of three closely related orphan receptors with no confirmed endogenous ligands, the two others being GPR6 and GPR12 [23, 24]. GPR3 is constitutively active and capable of signaling through G protein-dependent and -independent mechanisms [23, 24]. Previous studies have reported its involvement in a variety of pathophysiological conditions [23, 24]. Although an orphan receptor, GPR3 is phylogenetically most closely related to the cannabinoid receptors. Using β-arrestin2 recruitment assays, we recently found that CBD is an inverse agonist for GPR3 [25].

This study investigated whether CBD affected inflammatory responses of mouse primary glial cells stimulated by LPS, what signaling pathways were involved in the downstream effect of CBD and whether the effect of CBD on either astrocytes or microglia was mediated by GPR3. Our results demonstrated that CBD exerted potent anti‐inflammatory effects on both astrocytes and microglial cells by inhibiting pro-inflammatory cytokines and NF‐κB signaling pathways in vitro. The beneficial effect of CBD was also shown in vivo. Although no relationship of GPR3 with this effect of CBD was established, CBD may have therapeutic potential for neurodegenerative disorders, such as Multiple Sclerosis and Alzheimer’s disease, where microglia and astrocytes are important in the development of neuroinflammation.

## Methods and materials

### Reagents

CBD (Cat# 90080, Cayman Chemicals), Collagenase D (Cat# C7657, Sigma), Dispase (Cat# 17105–041, GibcoBRL), DNase I (Cat# 104159, Boehringer Mannheim), PPARγ antagonist-GW9662 (Cat# 1508, Tocris), LPS (Lipopolysaccharides from Escherichia coli 055:B5, Cat# L2880 or 055:B4, Cat# L2830, Sigma) and Percoll (Cat# 17-0891-02, GE-healthcare) were used in this study. All the antibodies for flow cytometry analysis were purchased from BioLegend unless otherwise stated.

### Mice

C57BL/6J (B6) mice were purchased from Jackson Lab. GPR3+/- mice with B6 background were acquired from the Mutant Mouse Regional Resource Center (MMRRC, stock ID: 011623-UNC) and re-derivatized by Charles River Lab. All animal studies conformed to the Association for Research in Vision and Ophthalmology statement on the use of animals in Ophthalmic and Vision Research and were approved by Institutional Animal Care and Use Committee (IACUC), University of Louisville (IACUC #20765). GPR3+/- were crossed to obtain GPR3+/+, GPR3+/-, and GPR3-/- (GPR3 KO). Genotyping was performed from tail on genomic DNA using DNeasy blood & tissue kit (QIAGEN, Cat# 69506), conventional PCR amplification and gel electrophoresis as previously described [19]. The reverse primer (GGAATTAAGCCCTGGTGGACCTAAC) and forward primers (TATCCACTCTCCAAGAACCATCTGG and GGGCCAGCTCATTCCTCCCACTCAT) were used to amplify a 506 bp and 358 bp segment of the wild type and Neo resistance cassette of the mutant mouse, respectively.

### Isolation and culture of primary CNS astrocytes and microglia

The astrocytes from fetus of B6 or GPR3-/- mice at postnatal day 2–4 were isolated and cultured following the method described by Schildge S et al. [26] with modification. In brief, under a stereo microscope (Leica), the olfactory bulbs and the cerebellum were removed and the plate-like structure of the cortex was peeled away from the brain. The meninges were dissected from the cortex hemispheres. Cortex hemispheres were cut into small pieces which were incubated with 2.5% trypsin in the water bath at 37°C for 30 min. Single cell suspension was collected after centrifugation at 300 x g for 5 min, vigorously pipetted, placed into a T25 culture flask pre-coated with 50 μg/ml of poly-D-lysine (PDL, Corning, Cat# 354210). The cells were cultured with the complete media (CM: 1640 medium containing 10% heat-inactivated fetal bovine serum, 1% Penicillin/Streptomycin and 0.1% 2-mercaptoethanol) at 37°C in the 5% CO_2_ incubator. The medium was changed every 2–3 days. After 7–8 days, when cells were confluent, the cell culture flask was shaken at 180 rpm for 30 min on an orbital shaker to remove microglia. The supernatant containing microglia was discarded. The remaining cells were further cultured for additional 2–3 days and then incubated with 10 ml of 0.0625% trypsin for 40min at 37°C. The detached cells were collected as astrocytes after centrifugation at 180×g for 5min and further expanded for future experiments at 37°C in the 5% CO_2_ incubator. The undetached cells were used as microglia [27] for immediate experiments.

**Fig 1:**
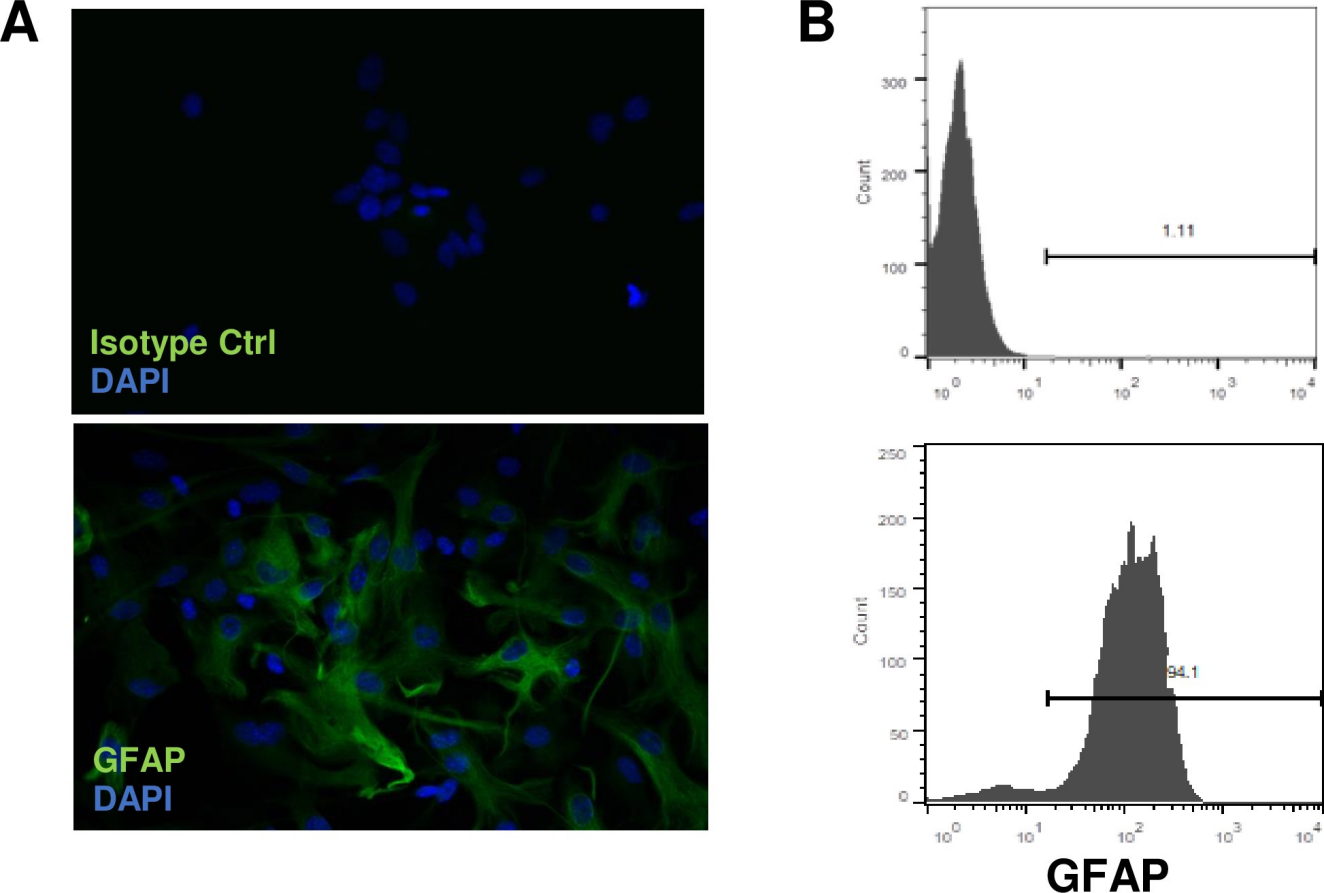
Characterization of brain astrocytes. Astrocytes from B6 mice were isolated as described in Materials and Methods. Before use, astrocytes were stained with mAb against GFAP (green) and nuclei with DAPI (blue) followed by immunofluorescence microscopy (A, 20 magnifications) and flow cytometry (B).

The purity of astrocytes was examined by staining the cells with anti- GFAP Ab followed by immunofluorescence microscopy and flow cytometry analysis (Fig 1). The ≥95% of GFAP positive cells with less than 5 passages were used for the experiments. Microglial cell purity was assessed by staining with anti–Iba-1 Ab (Cat# 10225, Abcam), showing that >98% were Iba-1+.

### Immunofluorescence

Astrocytes were cultured on the Nunc chamber slide (Thermo Fisher) till 60%-80% confluence. Cells were fixed with 4% paraformaldehyde for 15 min, blocked with 0.5% BSA for 30 min and permeabilized with 0.1%Triton-X 100 for 15 min. FITC-conjugated GFAP Ab (1:50 dilution, Cat# 53-9892-82, Invitrogen) was added to stain the cells, followed by DAPI staining for the nucleus. After mounting, astrocytes were examined by immunofluorescence microscopy (Nikon-E600). All procedures were conducted at room temperature (RT) [28].

### Cytokine detection by ELISA

Culture supernatants from astrocytes or microglia treated with medium plus CBD, or medium plus vehicle for 2 h followed by 0.1 μg/ml of LPS for an additional 4 h or 20 h were collected. Levels of IL-6 (Cat# DY406), TNF–α (Cat# DY410), IL-1β (Cat# DY401) and IL-10 (Cat# DY417) were determined by DuoSet ELISA kits following the manufacturer’s instruction (R&D System).

### Western blot analysis

To examine the levels of the phosphorylated form of the p65 NF-κB subunit, as well as activation of STAT3 and p42/44 MAPK after LPS stimulation in the presence or absence of CBD, astrocytes cultured in 100 mm plates were incubated with CBD at 10 μM for 2 h. Next, the cells were stimulated for 2 h with 0.1 μg/ml of LPS. The cells were washed with ice-cold PBS 3 times, and then the cells were lysed with cold RIPA buffer including protease and phosphatase inhibitors (Thermo) for 15 min on ice. Lysates were centrifuged at 12,000 g for 15 min at 4°C. The supernatants were collected for determining protein concentrations using Bradford assay (Sigma), aliquoted and stored at -20°C for further analysis.

A total of 20 μg protein samples were resolved by SDS-PAGE (8%) and transferred onto PVDF membranes (Sigma). The blotting was performed using the “Mini PROTEAN Tetra Electrophoresis System” (Bio-Rad) for 1.5 h at 85 Volts. Afterwards, the protein transferred membranes were incubated with non-fat dry milk in 0.05% (v/v) PBS/Tween for 1 h at RT, and then incubated overnight at 4°C with primary antibodies. Antibodies for β-actin (1:5,000, Cat# 4970S), NFκB/p65 (1:1,000 Cat# 8242T), p-NF-κB/p65 (1:1,000, Cat# 13346S), p44/42 MAPK (ERK1/2, 1:2000, Cat# 9102), p-p44/42 MAPK (1;2000, Cat# 4337), Stat3 (1:3,000, Cat# 9139T) and p-Stat3/Y705 (1:2,000, Cat# 4113S) were purchased from Cell Signaling Technology. After extensive wash with TBST, horseradish peroxidase-conjugated secondary goat anti-rabbit or anti-mouse antibodies (Santa Cruz) were applied for 1 h at RT, and the blots were extensively washed and visualized using an enhanced chemiluminescence detection kit (Millipore). The signal intensity was quantified with NIH ImageJ software. The intensity of the staining of β-actin was used as loading controls for data normalization. The ratio of phosphorylated versus general proteins was calculated.

### Flow cytometry

Aliquots of 5 × 10^5^ astrocytes or microglia were first blocked with CD32 and then stained with FITC-conjugated CD40 (Cat# 102905) and PE-conjugated CD11b (Cat# 101207) for microglial for 30 mins. For astrocytes, cells were further fixed, permeabilized 2 h with Cytofix/Cytoperm buffer (eBioscience), and incubated with FITC-conjugated anti-GFAP Ab (Cat# 53-9892-82, Invitrogen) or isotype control Ab for 30 min at 4°C. Data collection and analysis were performed on a FACScaliber flow cytometer using CellQuest software (BD, San Jose, CA, USA).

### LPS treatment in vivo and isolation of enriched astrocytes and microglia from brain

B6 mice (6 weeks-old) were intra-peritoneally (ip) injected with 10mg/kg or 75mg/kg of CBD (dissolved in1:1:8 of Ethanol, Tween 20 and PBS) or vehicle followed by 5mg/kg *of Escherichia coli* LPS (serotype 0111:B4) after 30 min. Naïve mice were ip given by vehicle and PBS. Four hours later, microglia and astrocytes were isolated from whole brain homogenates as previously described [29, 30] with some modifications. In brief, after anesthetization, mice were perfused with ice-cold PBS through the left ventricle and whole brains were collected. Brains were homogenized in digestion cocktail (mixture of 9.54 ml RPMI-1640, 50 μl of 10% collagenase D, 50 μl of 5 U ml^–1^ DNase I and 250 μl of 20% dispase). Resulting homogenates were passed through a 70 μm nylon cell strainer and then gently rocked at room temperature (RT) for 15 min. The suspensions were centrifuged at 300g for 7 min at 18 ^o^C, and the pellets were re-suspended in 70% isotonic Percoll at RT. A discontinuous Percoll density gradient was set up as follows: 70%, 50%, 35%, and 0% isotonic Percoll. The gradient was centrifuged for 20 min at 2000g, and astrocytes and microglia were collected from the interphase between the 70% and 35% Percoll layers. Cells were washed, re-suspended in sterile RPMI-1640 and immediately used for RNA extraction.

### Real-time PCR

Total RNA of astrocytes and microglial isolated from wild-type (WT) B6 mice or GPR3 KO mice was extracted using an RNeasy® Mini Kit (Qiagen). The RNA was reverse-transcribed into cDNA using a Moloney murine leukemia virus-RT kit (Invitrogen, Cat# 28025–013). Each cDNA sample was amplified for mouse interested genes using the GoTaq® qPCR and RT-qPCR systems (Promega, Cat# A6001) in Stratagene 3005 instrument. The QuantiTect primer QT00249732 against mouse GPR3 was purchased from Qiagen. Other primers used were: β-actin, 5’-ATCTACGAGGGCTATGCTCTCC (forward) and 5’-ACGCTCGGTCAGGATCTTCAT (reverse); IL-6, 5’-CCGGAGAGGAGACTTCACAG (forward) and 5’-TTCTGCAAGTGCATCATCGT (reverse); IL-1β, 5’-ACTCATTGTGGCTGTGGAGA (forward) and 5’-TTGTTCATCTCGGAGCCTGT (reverse) and TNF-α, 5’- CGTCGTAGCAAACCACCAAG (forward) and 5’-GGCAGAGAGGAGGTTGACTT (reverse). The expression of interested genes was determined using the comparative threshold cycle number and normalized to that of the internal β-actin control.

### Data analysis

Experiments were performed at least three times. Statistical analyses were performed using the unpaired Student’s t-test for two sets of data, and one-way ANOVA followed by Dunnett’s test for three or more means. Values determined to be significantly different from those of LPS treated group are marked with asterisks in the figures (*P < 0.05, **P < 0.01, ***P < 0.001).

## Results

### CBD inhibited IL-6 production by LPS-stimulated astrocytes

**Fig 2:**
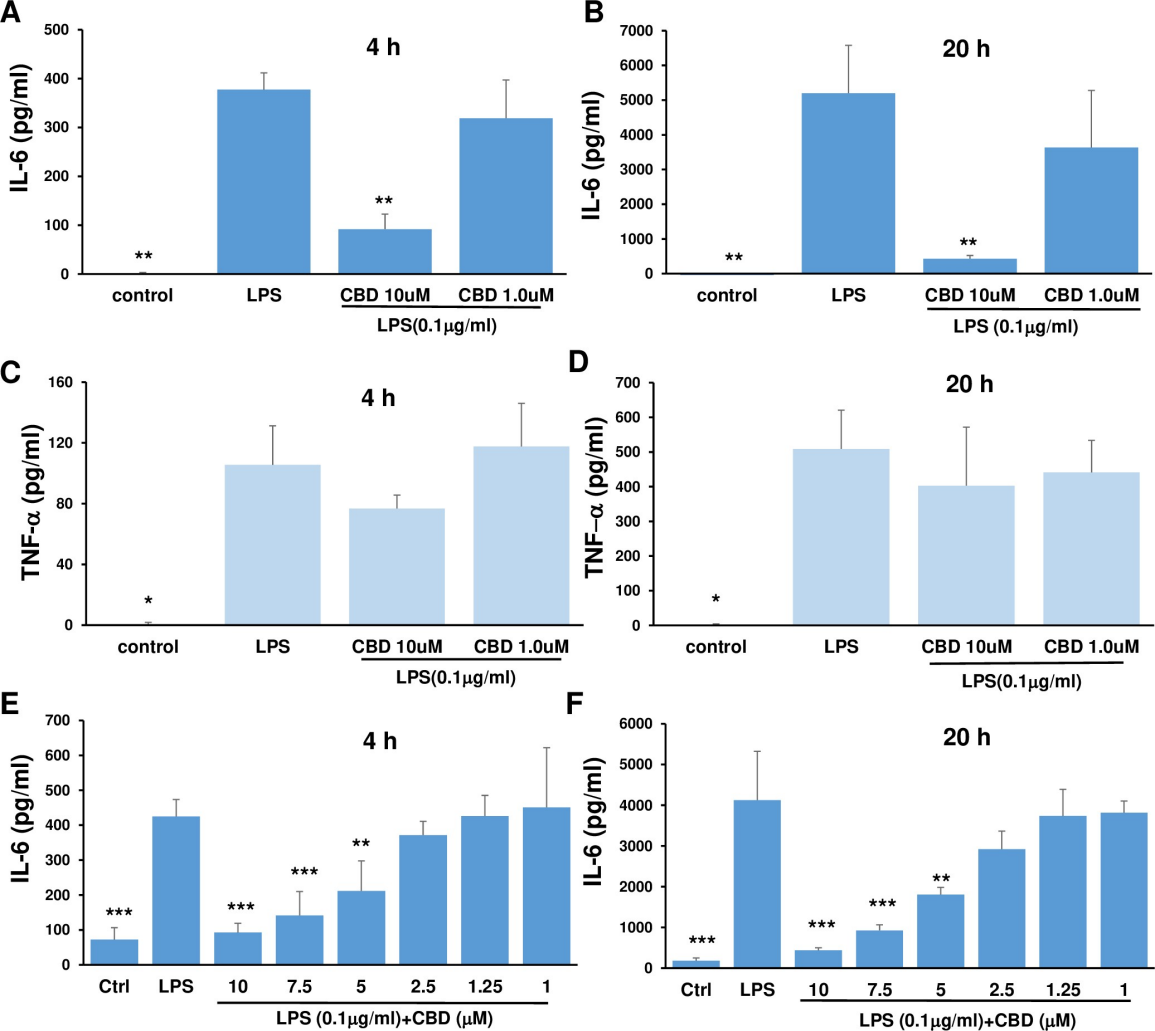
CBD inhibited IL-6 production by LPS-stimulated astrocytes. Astrocytes were incubated with medium and CBD in a 12-well plate for 2 h followed by LPS for an additional 4 h or 20 h, then the supernatants were collected for measurement of IL-6 (A, B, E, F) and TNF-α (C, D) by ELISA. The results were expressed as mean ± SEM. *p<0.05, **p < 0.01 and ***p<0.001 compared to LPS using one-way ANOVA followed by Dunnett’s test.

Astrocytes express toll-like receptors (TLRs) and produce cytokines in response to TLR ligands of pathogen-associated molecular patterns (PAMPs) and damage associated molecular patterns (DAMPs) [31, 32]. CBD has anti-inflammatory properties [6, 33]. To determine whether CBD can inhibit the inflammatory reaction of astrocytes to TLR4, we isolated mouse astrocytes from CNS cortex (Fig 1). The isolated astrocytes were treated with increasing doses of CBD for 2 h, followed by either an additional 4 or 20 h of stimulation with 0.1 μg/ml of LPS. As seen in Fig 2, astrocytes produced high levels of IL-6 and TNF-α as early as 4 h after stimulation with LPS. CBD at a high dose (10 μM) significantly inhibited IL-6 release at both time points (Fig 2A & 2B). In contrast, CBD did not inhibit TNF-α release at any time point (Fig 2C & 2D). This result suggested that CBD specifically inhibited IL-6 production by LPS-stimulated astrocytes. Moreover, a dose response curve of CBD between 1 and 10 μM was tested. It showed that the lowest concentration of CBD to inhibit LPS induced IL-6 was 5 μM (Fig 2E & 2F).

### CBD inhibited LPS stimulated phosphorylation of NF-κB and STAT3 pathways in astrocytes and the inhibition of IL-6 did not involve PPARγ

**Fig 3:**
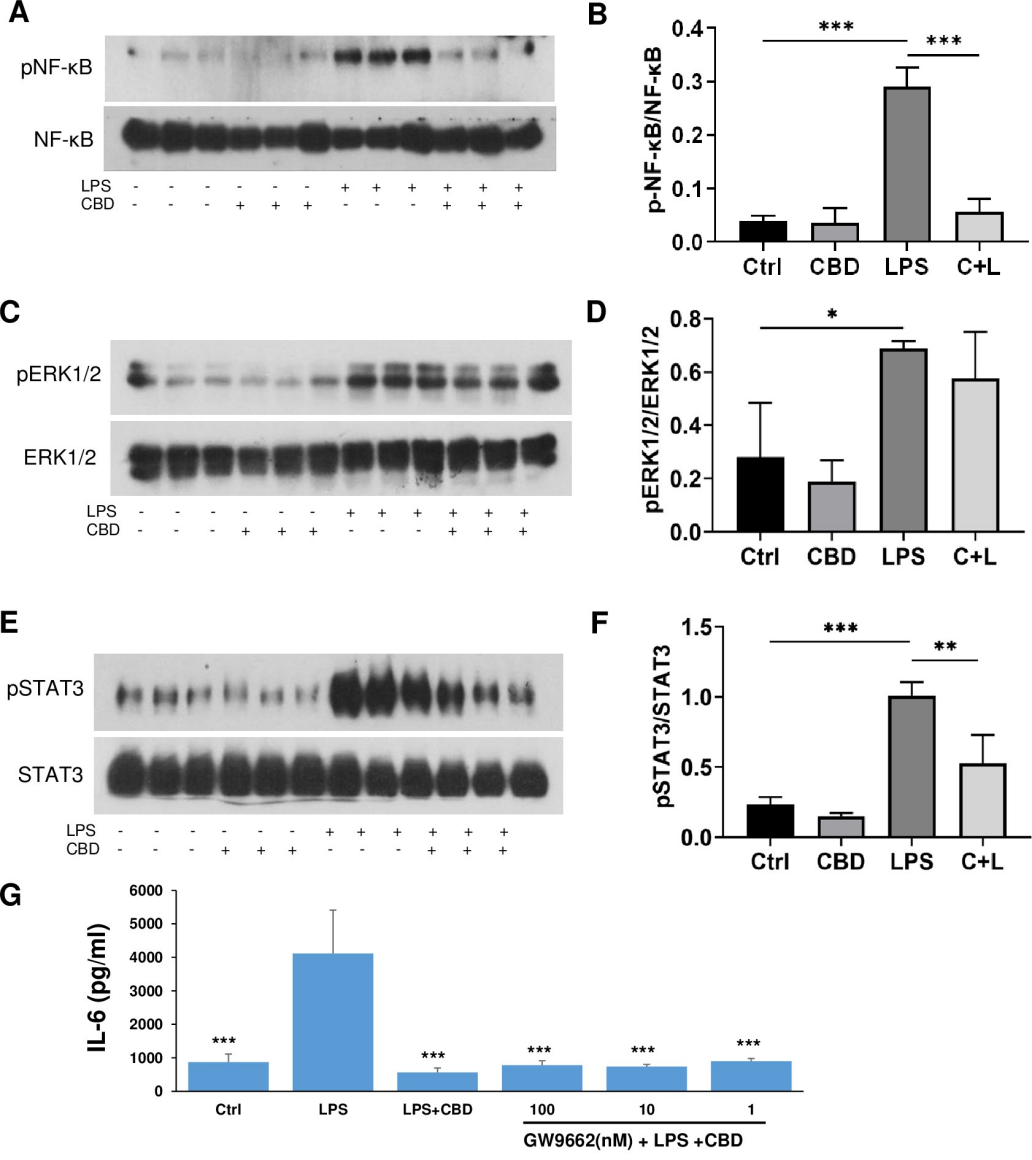
CBD reduced LPS-stimulated phosphorylation of NF-κB/p65 subunit and STAT3, but not MAP kinase in astrocytes and the inhibition of IL-6 did not involve PPARγ. Cells were pre-treated for 2 h with 10 μM of CBD followed by 2 h incubation with 0.1 μg/ml of LPS and lysed in RIPA buffer with proteinase and phosphatase inhibitors. Cell homogenates (20 μg of protein aliquots) were subjected to Western blot analysis using antibodies against pNF-κB/p65 (ser-536) subunit and the general form of NF-κB/p65(A, B), pERK1/2 and the general form ERK1/2 (C, D) and pSTAT3 and the general form of STAT3 (E, F). A, D, E show representative Western blots of each treatment of three replicates. B, D, F are bar graphs showing the average results of triplets of the ratio of phosphorylated NF-κB, ERK1/2 and STAT3 versus their general proteins. The data were analyzed by one-way ANOVA followed by Dunnett’s test. *p<0.05, **p<0.01, ***p<0.001 compared to LPS. F, G: Astrocytes were incubated with medium and increasing doses of GW9662 for 30 min followed by 10 μM CBD. After 2 h, 0.1 μg/ml of LPS was added and incubated for an additional 20 h, and then the supernatants were collected for measurement of IL-6 by ELISA. The results were expressed as mean ± SEM. ***p<0.001 compared to LPS using one-way ANOVA followed by Dunnett’s test.

We next investigated the signaling pathways involved in CBD inhibition of IL-6. NF-κB is a primary pathway involved in regulation of the expression of pro-inflammatory cytokine genes. The NF-κB p65-p50 protein complex is present in an inactive form in the cytoplasm. It has been reported in many cell types, including astrocytes, that LPS activation of TLR4 leads to MyD88 dependent phosphorylation of the NF-κB/p65 subunit that is then translocated to the nucleus [22]. We examined the expression of NF-κB/p65 and its phosphorylated form, p-NF-κB/p65, in the LPS stimulated astrocytes in the presence or absence of CBD by Western blot. As shown in Fig 3A and 3B, LPS (0.1 μg/ml) stimulation resulted in an increase in p-NF-κB/p65 and pretreatment with CBD completely inhibited phosphorylation of NF-κB/p65.

LPS has been shown to activate not only NF-κB signaling but also members of the mitogen-activated protein kinase (MAPK) family, including extracellular signal–regulated kinases (ERK1/2), c-Jun amino terminal kinases (JNKs), and p38 [34, 35]. These kinases ultimately control the activity of transcription factors that modulate the expression of inflammatory molecules including TNF-α, IL-1α, IL-1β and IL-6 [36]. As seen in Fig 3C and 3D, 2 h after stimulation with LPS, phosphorylation of ERK1/2 was significantly increased and CBD did not prevent the LPS-induced increase in phosphorylation. LPS stimulation has also been demonstrated to potentiate the phosphorylation of STAT3 in astrocytes [37, 38]. As shown in Fig 3E and 3F, STAT3 phosphorylation was enhanced in astrocytes after LPS stimulation and the effect was significantly attenuated by CBD. PPARγ has been reported to be involved in CBD neuroprotection [17]. We pre-treated astrocytes with increasing doses of GW9662, a potent PPARγ antagonist, prior to CBD and LPS. As shown in Fig 3G, GW9962 did not reverse the inhibitory effect of CBD on IL-6 production. Thus, our results demonstrated that CBD significantly inhibited LPS-stimulated phosphorylation of both NK-κB and STAT3, but not ERK1/2. In addition, PPARγ was not a target for CBD inhibition of IL-6 in LPS-stimulated astrocytes.

### The inhibition of CBD on IL-6 production by LPS-stimulated astrocytes was not dependent on GPR3

**Fig 4:**
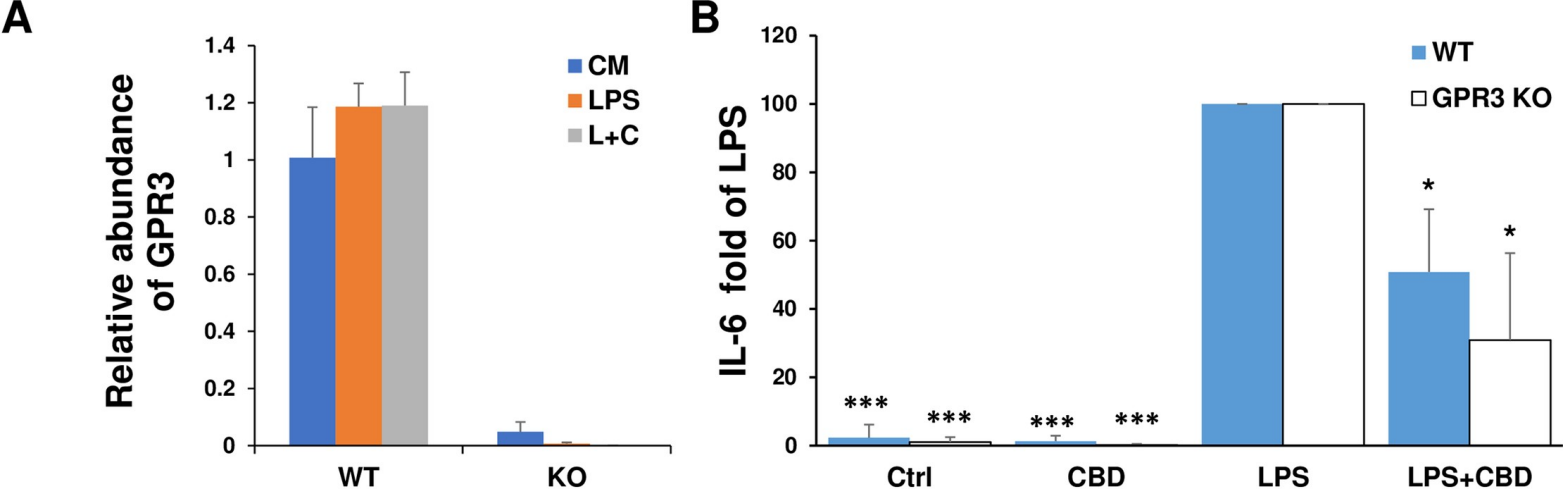
Isolated astrocytes expressed GPR3 and knockout of GPR3 did not reverse CBD inhibition of IL-6 production by LPS stimulated astrocytes. (A) The expression of GPR3 in astrocytes at mRNA levels. Astrocytes isolated from WT B6 mice or GPR3 KO mice were incubated with medium, 0.1 μg/ml LPS, or 10 μM CBD for 2 h followed by 0.1 μg/ml LPS (L+C). After 20h, mRNA levels of GPR3 were determined by RT-PCR and the relative abundance of GPR3 was expressed compared to housekeeping gene β-actin. (B) Astrocytes isolated from WT or GPR3 KO mice were incubated with medium, 0.1 μg/ml LPS, 10 μM CBD or CBD for 2 h followed by LPS. After 20h, the supernatants were collected for measurement of IL-6 by ELISA. The fold changes of IL-6 in different cell culture supernatants compared to supernatants in LPS stimulated astrocytes (as 100%) were calculated from 3 individual experiments. *p< 0.05 and *** *p*<0.001 compared to LPS using one-way ANOVA followed by Dunnett’s test.

Having defined the signaling pathways of inhibition of CBD on LPS stimulated astrocytes, we wanted to explore CBD action at the receptor level. There are two major cannabinoid receptors CB1 and CB2; the former is highly expressed in the brain and the latter is mostly restricted to immune cells [39]. CBD has low affinity to CB1 and CB2. GPR3 is predominantly expressed in mammalian brain [24] and CBD is an inverse agonist to GPR3 [25]. Thus, we examined whether the inhibition of CBD in IL-6 production by LPS-stimulated astrocytes is mediated by GPR3. We first examined the expression of GPR3 in isolated astrocytes from WT B6 mice and GPR3 KO mice by RT-PCR. As seen in Fig 4A, the mRNA level of GPR3 was constitutively expressed in purified WT astrocytes but not astrocytes isolated from GPR3 KO mice. Neither LPS stimulation nor the combination of LPS and CBD altered the level of expressed GPR3. Further, we cultured WT astrocytes and GPR3 KO astrocytes and stimulated them with LPS in the presence or absence of CBD. Fig 4B showed the change of IL-6 in culture supernatants of WT and GPR3KO astrocytes from three individual experiments using LPS induced IL-6 as 100%. Both WT and GPR3 KO astrocytes responded to LPS, and inhibition of IL-6 production by CBD was observed in both astrocytes. Our data indicate that CBD-induced inhibition of IL-6 release is not dependent on GPR3 in primary mouse astrocytes.

### GPR3 on microglia did not mediate the inhibition of CBD on cytokine production by LPS

**Fig 5:**
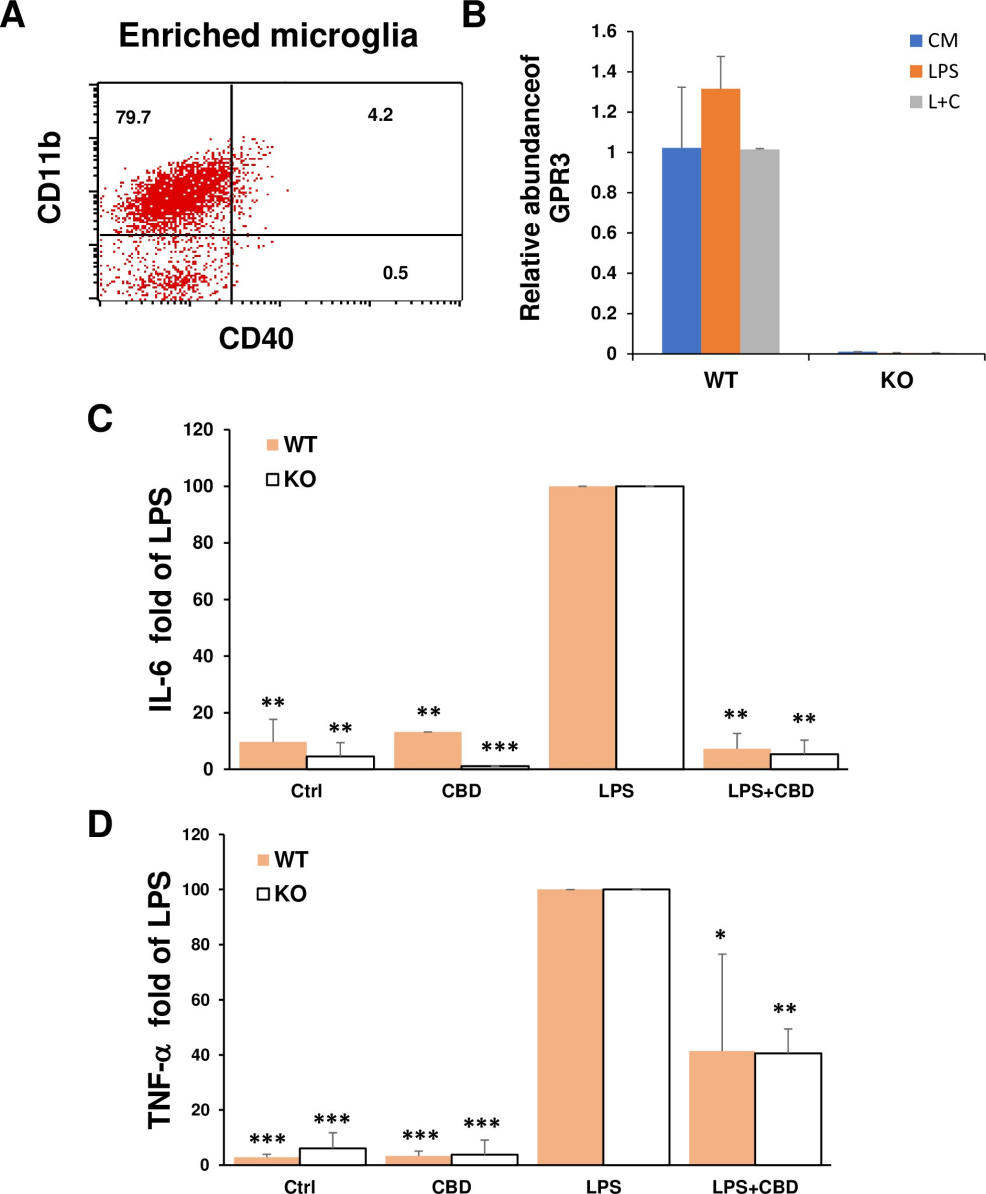
Microglia expressed GPR3 but the knockout of GPR3 did not prevent CBD inhibition of cytokine production after LPS stimulation. (A) Enriched resting microglial cells isolated as described in the Methods section were characterized as CD11b+ CD40- by flow cytometry. (B) The expression of GPR3 in microglia at mRNA levels. Microglia isolated from WT B6 mice or GPR3 KO mice were incubated with medium, 0.1 μg/ml LPS, or 10 μM CBD for 2 h followed by 0.1 μg/ml LPS (L+C). After 20h, mRNA levels of GPR3 were determined by RT-PCR and the relative abundance of GPR3 compared to housekeeping gene β-actin. (C, D) Microglial isolated from WT and GPR3 KO mice were incubated with medium or CBD for 2 h followed by LPS for an additional 20 h; the supernatants were collected for measurement of IL-6 and TNF-α by ELISA. The fold change of IL-6 (C) and TNF-α (D) in different cell culture supernatants compared to the supernatants in LPS stimulated microglia (as 100%) were calculated from 3 individual experiments. *p< 0.05, **p< 0.01 and *** *p*<0.001 compared to LPS using one-way ANOVA followed by Dunnett’s test.

It has also been reported that CBD inhibits cytokine production of LPS-stimulated microglial cell lines. This inhibition was not mediated by CB1, CB2, and abn-CBD-sensitive receptors [40]. To determine whether GPR3 is a receptor for CBD in primary microglia, we isolated microglial cells which were characterized as CD11b+CD40- by flow cytometry (Fig 5A) and examined the expression of GPR3 on these cells. The results in Fig 5B showed that GPR3 was constitutively expressed in WT but not GPR3 KO microglia. This expression was not altered by LPS stimulation or the combined treatment of LPS and CBD. The data in Fig 5C and 5D compared the change of IL-6 and TNF-α in culture supernatants of WT and GPR3KO microglia from three individual experiments using LPS-induced IL-6 or TNF-α as 100%. The inhibition of CBD on IL-6 and TNF-α release stimulated by LPS in WT microglia was not abrogated in microglia isolated from GPR3 KO mice (Fig 5C & 5D). These data demonstrate that GPR3 is not involved in CBD-induced inhibition of IL-6 and TNF-α release from LPS-stimulated primary microglia cells.

### In vivo CBD reduced LPS induced IL-1β and TNF-α in astrocytes and microglia

**Fig 6:**
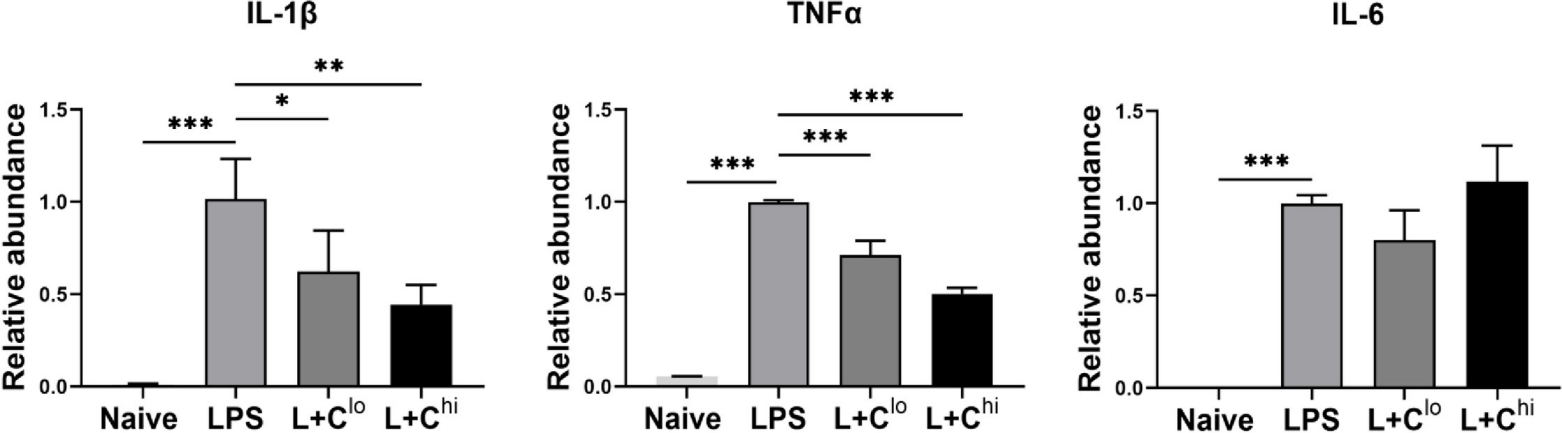
In vivo CBD reduced LPS induced IL-1β and TNF-α in astrocytes and microglia. B6 mice were randomly separated into three groups for intraperitoneal (ip) injection of vehicle, 10mg/kg CBD or 75mg/kg of CBD. After 30 min, all mice were given 5mg/kg of LPS ip. Naïve mice received both vehicle and PBS. Four hours later, astroglia including astrocytes and microglia were isolated from brain homogenates. mRNA levels of IL-1β, TNF-α and IL-6 were determined by RT-PCR and the relative abundance of these genes were expressed compared to housekeeping gene β-actin. Five brains per group were pooled for one experiment. Data from two independent experiments is presented. Means ± SEM are shown. *p<0.05, **p<0.01 and ***p<0.001 compared to LPS group using one-way ANOVA followed by Dunnett’s test.

B6 mice were ip injected with CBD 30 min prior to LPS. As shown in Fig 6, both low and high concentrations of CBD significantly inhibited mRNA expression of IL-1β and TNF-α but not IL-6 in astrocytes and microglia collected 4 h after LPS administration. These results confirmed that the anti-inflammatory effects of CBD were observed both in vitro and in vivo.

## Discussion

Astrocytes respond to CNS injury and disease by producing many different cytokines and inflammatory mediators [20, 21]. In this study, we investigated the effects of CBD on the responses of primary mouse astrocytes to LPS, a ligand that binds TLR4, one of the most common receptors recognizing PAMPs/DAMPs [31, 32]. We discovered for the first time that CBD was able to inhibit LPS-stimulated IL-6 release from primary mouse astrocytes. Our data are consistent with a previous report that CBD inhibits IL-6 release from BV2 cells, a mouse microglial cell line [40]. Using primary mouse microglial cells, we replicated this previous finding. Since IL-6 is an important cytokine in neuroinflammation, our results support the notion that CBD may be useful in treating some neurological disorders.

In the present study, LPS-stimulated TNF-α release from mouse astrocytes was not significantly affected by CBD. However, a previous publication demonstrated that CBD inhibited β amyloid (Aβ)-stimulated TNF-α release from cultured rat astrocytes [17]. This discrepancy could be due to the different models (LPS vs Aβ) used to study the effect of CBD. However, both our data and the previous report support the concept that CBD may have therapeutic potential for neuroinflammation [17, 40, 41]. Most importantly, our in vivo study showed that CBD did inhibit LPS induced IL-1β and TNF-α in astroglial and microglial cells. Although CBD inhibited IL-6 production in both LPS-stimulated astrocytes and microglia in vitro, it did not reduce IL-6 expression in both cell types in vivo 4 h after LPS injection. The inhibition of CBD on different pro-inflammatory cytokines might be time dependent. In a recent study in a mouse model of depression, CBD reduced both serum and prefrontal cortex IL-6, but not TNF-α,12 h after LPS administration [42]. It seems that IL-1β and TNF-α may respond earlier than IL-6 to CBD inhibition.

Interestingly, we detected GPR3 mRNA in highly enriched mouse astrocytes. The protein level of GPR3 was not studied due to the lack of a validated antibody specific for mouse GPR3. The inhibitory effects of CBD on LPS-stimulated IL-6 release in WT astrocytes were also seen in astrocytes isolated from GPR3 deficient mice. These results suggest that CBD inhibition of IL-6 is not mediated by GPR3; similar results were with primary microglia. Therefore, the receptor involved in CBD inhibitory action on pro-inflammatory cytokine production remains to be explored in both mouse astrocytes and microglia.

CBD has many potential molecular targets [1, 43]. In addition to GPR3, several other targets, including CB1, CB2 and abn-CBD receptors have been excluded for CBD-induced inhibition of IL-6 in a microglia cell line [40]. PPARγ and Wnt3 are two other possible targets for the inhibitory effects of CBD on cytokine release from astrocytes; both of these proteins have been shown to be involved in the neuroprotective and CNS anti-inflammatory actions of CBD in previous publications [17, 41]. Our data excluded the possibility that PPARγ was the target of CBD on IL-6 production by LPS-stimulated primary astrocytes and is similar to previous reports that not all anti-inflammatory effects of CBD involves PPAPγ [42, 44]. It is known that both STAT3 and NF-κB pathways promote the production of IL-6 in response to LPS [45]. In our system, both pSTAT3 and pNF-κB were stimulated by LPS and addition of CBD inhibited the phosphorylation of STAT3 and NF-κB. However, due to the technical difficulty of transfecting primary astrocytes, we are unable to establish a robust over expression of STAT3 and NF-κB in our models. Therefore, we cannot say conclusively that STAT3 and NF-κB are involved in CBD-induced inhibition of IL-6 release, only that there is an association between CBD-induced inhibition of IL-6 release and CBD-induced inhibition of STAT3 and NF-κB phosphorylation. Nuclear factor IL-6 (NF-IL-6) is a major regulator of IL-6 transcription and is known to interact with NF-κB [46, 47]. Since we have shown that CBD inhibits both IL-6 release and NF-κB phosphorylation, in the future we plan to study whether NF-IL-6 is involved in the actions of CBD.

In summary, our results show that CBD inhibited the LPS activation of pro-inflammatory signaling in primary astrocytes. In addition, we examined and excluded the involvement of the novel CBD receptor-GPR3 in inhibition of LPS-stimulated inflammatory responses of astrocytes and microglia. Most importantly, our current data, together with previously published results by other groups [17, 40, 41], suggests that CBD inhibition of pro-inflammatory signaling networks in astrocyte and microglial cells may have therapeutic potential in CNS neuroinflammatory diseases.

## Supporting information

S1 Raw image (TIF)
