# Supplementary figures and images for: Studies of involvement of G-protein coupled receptor-3 in cannabidiol effects on inflammatory responses of mouse primary astrocytes and microglia
Source: PLoS One. 2021 May 13;16(5):e0251677. doi: 10.1371/journal.pone.0251677 (PMC8118243; doi:10.1371/journal.pone.0251677)

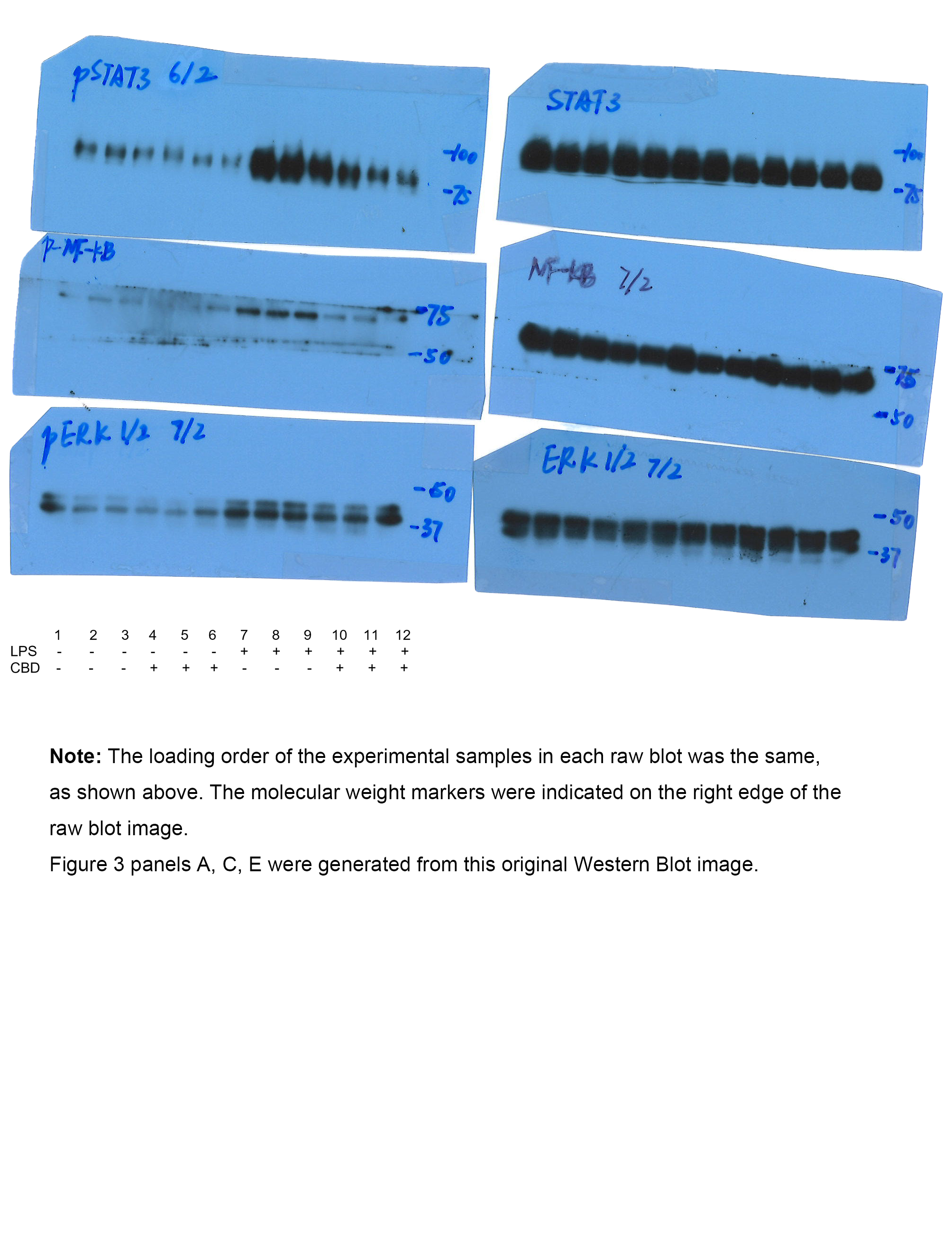

Supplement: S1 Raw image — (TIF) [file pone.0251677.s001.tif]
